# Selenium nanoparticles and wheat straw biochar synergistically alleviate combined drought–heat stress in wheat (*Triticum aestivum* L.) by modulating antioxidant defense, photosynthetic efficiency, and ion homeostasis

**DOI:** 10.1080/15592324.2026.2681236

**Published:** 2026-07-07

**Authors:** Hina Firdous, Abbas Shoukat, Muhammad Mubashar Zafar, Huma Saleem, Rabia Faridi, Arfan Ali, Waseem Hassan, Sezai Ercisli, Rafiuddin Rafiuddin, Muhammad Fuad Anshori, Majed Alotaibi, Mahmoud F. Seleiman

**Affiliations:** a State Key Laboratory of Crop Stress Resistance and High-Efficiency Production, College of Plant Protection, Northwest A&F University, Yangling, Shaanxi, China; b Institute of Plant Nutrition and Soil Science, Kiel University, Kiel, Germany; c Department of Plant Breeding and Genetics, UAF, Faisalabad, Pakistan; d FB Genetics, Four Brothers Group, Lahore, Pakistan; e Soil and Water Testing Laboratory Lodhran, Punjab, Pakistan; f Department of Horticulture, Faculty of Agriculture, Ataturk University, Erzurum, Turkey; g Agronomy Department, Faculty of Agriculture, Hasanuddin University, Makassar, Indonesia; h Plant Production Department, College of Food and Agriculture Sciences, King Saud University, Riyadh, Saudi Arabia

**Keywords:** Selenium nanoparticles, wheat straw biochar, combined abiotic stress, *Triticum aestivum*, antioxidant defense, photosynthesis, ion homeostasis, reactive oxygen species

## Abstract

Combined drought and heat stress (DS+HS) represents one of the most devastating multi-factorial constraints on global wheat (*Triticum aestivum* L.) production, imposing synergistic physiological and molecular damage that far exceeds the effects of each stress applied individually. Emerging evidence suggests that selenium nanoparticles (Se NPs) and biochar independently confer abiotic stress tolerance in cereal crops; however, their combined soil application under concurrent DS+HS in wheat has not been previously investigated. The present study was conducted at the wire house facility of the Four Brothers Group (FBG), Lahore, Pakistan, using soil collected from Faisalabad, to evaluate the individual and combined effects of soil-applied Se NPs (50 mg kg^−1^) and wheat straw biochar (2% w/w) on wheat cv. FBG-1800 subjected to drought (30% field capacity) and heat stress (42 °C, 6 h d^−1^) in a completely randomized design with three replications. Combined DS+HS severely impaired growth, photosynthetic function, and grain yield, while markedly elevating oxidative damage markers and disrupting ionic homeostasis. Notably, combined Se NPs + biochar application produced superior synergistic amelioration relative to either amendment alone, restoring net photosynthetic rate and grain yield to approximately 89%–90% and 88% of control values, respectively, alongside significant reductions in oxidative stress indicators. Gene expression analysis confirmed marked upregulation of key stress-responsive genes (TaSOD1, TaDREB2, TaHSP70, TaNHX1) and coordinated antioxidant enzyme activation. These findings establish that soil co-application of Se NPs and biochar is an effective, agronomically feasible strategy to sustain wheat productivity under the increasingly prevalent combined DS+HS conditions in Pakistan's irrigated cropping systems.

## Introduction

Wheat (*Triticum aestivum* L.) is the world's most widely cultivated cereal crop, providing approximately 20% of daily caloric and protein intake for the global human population. It constitutes the foundation of food security across South Asia, the Middle East, and North Africa.^1^ In Pakistan, wheat is cultivated on approximately 9.0 million hectares, accounting for over 60% of total cereal area, and contributes substantially to national agricultural GDP and rural livelihoods.^2^ However, Pakistani wheat production faces escalating and increasingly concurrent abiotic stressors, principally drought and high temperature, both of which are intensifying under contemporary climate change trajectories.^3^ Summer temperatures in major wheat-growing districts of Punjab regularly exceed 38 °C–42 °C during the critical grain-filling period (March–May). Concurrently, erratic monsoon patterns and groundwater depletion impose episodic to chronic drought stress throughout the growing season.^4,5^

Drought stress impairs wheat through three interlocking mechanisms: osmotic stress arising from reduced soil water potential; ionic imbalance from disrupted mineral absorption; and secondary oxidative stress from excessive accumulation of reactive oxygen species (ROS), including superoxide anion (O₂•^−^), hydrogen peroxide (H₂O₂), and hydroxyl radicals (•OH). These ROS damage cellular membranes, proteins, lipids, and nucleic acids^6,7^ Heat stress, acting independently, disrupts photosynthesis through PSII inactivation, Rubisco denaturation, and thylakoid membrane destabilization. It also impairs reproductive development, including pollen viability and grain set, and triggers protein misfolding cascades that overwhelm the cellular chaperone system.^8^ When drought and heat co-occur—as is increasingly the case in Pakistan's irrigated wheat belt—their combined impact is synergistic rather than simply additive. This combination creates a physiological dilemma: heat demands stomatal opening for transpirational cooling, while drought demands stomatal closure to conserve water, simultaneously maximizing damage from both stressors.^9‐11^

Against this challenge, nanomaterial-based agricultural interventions have attracted considerable recent interest. Selenium nanoparticles (Se NPs) represent a particularly promising avenue owing to selenium's dual role as an essential micronutrient at low concentrations and a potent antioxidant signaling molecule.^12^ Selenium activates key antioxidant enzyme systems—including superoxide dismutase (SOD), catalase (CAT), ascorbate peroxidase (APX), and glutathione peroxidase (GPX)—while also modulating ABA-mediated stomatal responses, osmolyte biosynthesis, and ion transporter gene expression.^13^ Nano-formulation of selenium confers superior bioavailability and reduced phytotoxicity relative to ionic selenate or selenite, and enhances rhizosphere interactions that improve uptake efficiency.^14^ Recent studies confirm that Se NPs at optimized concentrations (30–75 mg kg^−1^ soil) significantly enhance wheat tolerance to individual drought and heat stress through antioxidant defense activation, improved photosynthetic pigment stability, and modulation of stress-responsive gene expression.^14,15^

Biochar—the carbon-rich solid product of biomass pyrolysis under limited oxygen conditions—has emerged as a soil amendment with multifaceted benefits for plant stress tolerance.^16^ Wheat straw biochar, abundantly available as an agricultural by-product in Pakistan, significantly improves soil water retention capacity, cation exchange capacity (CEC), and pore structure, thereby alleviating drought-induced water deficit.^17^ Biochar also modulates soil pH, reduces heavy metal bioavailability, and improves rhizosphere microbial community structure, all of which facilitate nutrient uptake under stress conditions.^18^ At the plant physiological level, biochar application has been shown to enhance chlorophyll stability, osmolyte accumulation, and membrane integrity under combined abiotic stresses in wheat and other cereals.^19‐21^

Despite the promising individual profiles of Se NPs and biochar, no study to date has examined their combined soil application under concurrent drought and heat stress in wheat. Furthermore, their synergistic potential at the molecular gene expression level remains unexplored. The present study was therefore designed to address three specific gaps: (i) characterization of the synergistic physiological and biochemical damage imposed by combined DS+HS on wheat cv. FBG-1800; (ii) evaluation of individual and combined Se NPs and biochar soil application across growth, photosynthetic, oxidative, ionic, and yield parameters; and (iii) elucidation of the molecular mechanisms underlying Se NPs + biochar synergy through quantitative expression analysis of key stress-responsive genes.

The specific objectives of this study were: (1) to quantify the individual and combined effects of drought and heat stress on growth, photosynthesis, oxidative damage, antioxidant defense, ion homeostasis, and yield; (2) to assess the efficacy of soil-applied Se NPs (50 mg kg^−1^), wheat straw biochar (2% w/w), and their combination in alleviating combined DS+HS; (3) to characterize the expression of key stress-responsive genes (*TaSOD1*, *TaCAT1*, *TaAPX*, *TaDREB2*, *TaHSP70*, *TaNHX1*) under all treatment combinations; and (4) to propose mechanistic pathways explaining the observed synergistic effects of Se NPs and biochar on multi-stress tolerance in wheat.

## Materials and methods

### Plant material and experimental site

Seeds of wheat (*Triticum aestivum* L. cv. FBG-1800)—a medium-duration, high-yielding cultivar widely grown in Punjab, Pakistan—were obtained from the Four Brothers Group (FBG), Lahore, Pakistan. FBG-1800 was selected on the basis of its commercial significance in the Punjab Uniform Wheat Yield Trial (PUWYT) and its documented sensitivity to combined abiotic stresses, which allowed clear detection of treatment effects. Seeds were surface-sterilized in 1% sodium hypochlorite (NaOCl) solution for 10 min, rinsed five times with sterile distilled water, and air-dried prior to sowing. All experiments were conducted in the wire house facility of FBG, Lahore, Pakistan, under controlled environmental conditions (16/8 h photoperiod; PAR 600 µmol m^−2^ s^−1^; baseline temperature 25/20 °C d/night; 60% ± 5% relative humidity).

Plants were grown in plastic pots (30 cm diameter × 35 cm height; soil volume approximately 24.7 L per pot) containing 8 kg of sterilized sandy loam soil (pH 7.4; EC 0.38 dS m^−1^; organic matter 0.72%; total N 0.048%; available P 8.4 mg kg^−1^; available K 142 mg kg^−1^) collected from agricultural fields in Faisalabad. Five seeds were sown per pot and thinned to three uniform seedlings per pot at 10 d after sowing to ensure consistent plant density across all experimental units. Soil was sterilized by autoclaving at 121 °C for 1 h on three consecutive days. Basal fertilization was applied at planting: N at 120 kg ha^−1^ (as urea), P₂O₅ at 90 kg ha^−1^ (as diammonium phosphate), and K₂O at 60 kg ha^−1^ (as sulphate of potash), converted to pot equivalents based on surface area.

### Synthesis and characterization of selenium nanoparticles

Selenium nanoparticles (Se NPs) were synthesized by green biological reduction following the method of Pyrzynska and Sentkowska^22^ with minor modifications. Briefly, 100 mL of aqueous sodium selenite (Na₂SeO₃; 1 mM; Sigma-Aldrich, USA) solution was mixed with 10 mL of *Azadirachta indica* (neem) leaf extract under continuous magnetic stirring at 60 °C for 4 h in darkness. The color change from colorless to brick-red confirmed Se NP formation. The suspension was centrifuged at 12,000 rpm for 15 min, washed three times with distilled water and once with absolute ethanol, and freeze-dried at −50 °C for 48 h (Freezone 2.5, Labconco, USA). Physicochemical characterization was performed using: (i) X-ray diffraction (XRD; Bruker D8 Advance, Germany) to confirm crystalline structure; (ii) transmission electron microscopy (TEM; JEOL JEM-2100, Japan) for morphology and size determination; (iii) dynamic light scattering (DLS; Zetasizer Nano ZS, Malvern Panalytical, UK) for hydrodynamic diameter and polydispersity index (PDI); and (iv) Fourier-transform infrared spectroscopy (FTIR; PerkinElmer Spectrum 100, USA) for functional group identification. Mean particle size was 28.4 ± 3.2 nm, zeta potential −24.6 ± 1.8 mV (confirming colloidal stability), and PDI 0.18 (indicating narrow size distribution).

### Preparation and characterization of wheat straw biochar

Wheat straw biochar was produced by slow pyrolysis of locally collected wheat straw at 500 °C for 2 h under limited oxygen conditions using a laboratory muffle furnace (Nabertherm L 9/11, Germany), following the protocol of Haider et al. ^17^ Biochar was ground and sieved to pass a 2-mm mesh before application. Physicochemical properties were determined as follows: pH (1:10 biochar:water suspension; glass electrode pH meter, Mettler-Toledo); electrical conductivity (EC; same suspension); surface area by BET analysis (Quantachrome NOVAe 2200, USA); elemental composition (C, H, N, O) by CHNS analyzer (Elementar Vario EL III, Germany); and cation exchange capacity (CEC) by ammonium acetate method.^23^ Biochar characteristics: pH 9.1; EC 1.24 dS m^−1^; C content 62.4%; BET surface area 186.3 m² g^−1^; CEC 42.6 cmolc kg^−1^; H/C ratio 0.28 (confirming high aromaticity and stability).

### Experimental design and treatments

The experiment was laid out in a completely randomized design (CRD) with eight treatment combinations and three biological replications (*n* = 3) per treatment, totaling 24 experimental units (three plants per pot). Treatments are described in Table 1.

**Table 1.** Treatment design for Se NPs and biochar application under drought and heat stress in wheat.

Code	Treatment	Full description	Stress applied
T1	Control	No stress; distilled water; no amendments	None
T2	Drought stress (DS)	Water withheld at 30% field capacity for 21 d	Drought
T3	Heat stress (HS)	42 °C, 6 h d^−1^ (09:00–15:00 h) for 14 d	Heat
T4	DS+HS	Combined drought (30% FC) + heat (42 °C)	Drought + Heat
T5	Se NPs	Soil application of Se NPs at 50 mg kg^−1^; no stress	None
T6	Biochar	Soil application of wheat straw biochar at 2% w/w; no stress	None
T7	DS+HS+Se NPs	Combined stress + Se NPs at 50 mg kg^−1^ soil	Drought + Heat
T8	DS+HS+Biochar+Se NPs	Combined stress + Se NPs (50 mg kg^−1^) + Biochar (2% w/w)	Drought + Heat

CRD = completely randomized design; Se NPs = selenium nanoparticles; FC = field capacity; DS = drought stress; HS = heat stress. *n* = 3 per treatment.

Se NPs were incorporated into the soil at 50 mg kg^−1^ (soil dry weight basis) by thoroughly mixing with the top 15 cm of pot soil one week before sowing. This concentration was selected based on previous studies reporting optimal stress-ameliorative effects of Se NPs in wheat and other cereals within the 30–75 mg kg^−1^ range^14,15^ with 50 mg kg^−1^ consistently producing significant physiological improvements without phytotoxic effects. Wheat straw biochar was incorporated at 2% (w/w; 160 g per pot) by mixing uniformly with the entire soil volume at pot preparation. This rate was chosen based on established literature demonstrating significant improvements in soil water retention, CEC, and plant stress tolerance in wheat at 1.5%–2.5% w/w.^17,21^ It is acknowledged that a full dose-response evaluation was not within the scope of the present study; optimization of application rates across soil types and stress intensities is identified as a priority for future investigation.

Drought stress was imposed from day 30 post-sowing, corresponding to the flag leaf stage (GS 37 on the Zadoks scale), by reducing irrigation to achieve and maintain 30% of field capacity (FC). Soil moisture was monitored daily by the gravimetric pot-weighing method for 21 consecutive days. FC was pre-determined for each soil lot using the standard pressure plate method. Heat stress was applied simultaneously with drought stress from day 30 (GS 37), in a programmable temperature-controlled growth chamber (Conviron CMP6050, Canada) set to 42 °C for 6 h d^−1^ (09:00–15:00 h) for 14 consecutive days, simulating peak summer temperatures during the grain-filling period in Punjab, Pakistan. This growth stage was deliberately targeted as it coincides with the most stress-sensitive phase of wheat development, encompassing anthesis and early grain filling. Control plants were irrigated at 80% FC and maintained at 25 °C throughout the experimental period.

#### Growth and biomass parameters

At harvest (day 70 post-sowing, physiological maturity), plant height (cm) was measured from soil surface to the tip of the tallest tiller using a measuring tape. Root length (cm) was measured after carefully washing roots free of soil under running water. Shoot fresh weight (SFW, g plant^−1^) was recorded immediately after harvest on a digital analytical balance (Shimadzu ATX224, Japan; 0.0001 g precision); shoot dry weight (SDW, g plant^−1^) was determined after oven-drying at 70 °C for 72 h (Memmert UFB 400, Germany). Leaf relative water content (LRWC, %) was determined on the penultimate fully expanded leaf using: LRWC = [(FW − DW)/(TW − DW)] × 100, where TW = turgid weight after floating on distilled water in darkness for 4 h.^24^ Root-to-shoot ratio was calculated as dry weight basis. Stem diameter (mm) was measured at the first internode using a digital Vernier caliper.

### Photosynthetic gas exchange and pigment analysis

Net photosynthetic rate (Pn, µmol CO₂ m^−2^ s^−1^), stomatal conductance (gs, mol H₂O m^−2^ s^−1^), transpiration rate (E, mmol H₂O m^−2^ s^−1^), and intercellular CO₂ concentration (Ci, µmol mol^−1^) were measured on the penultimate fully expanded leaf of three plants per pot between 09:00–11:00 h, using a portable infrared gas analyzer (LI-6400XT, LI-COR Biosciences, USA) at ambient CO₂ (400 µmol mol^−1^) and PAR of 1000 µmol m^−2^ s^−1^. Water use efficiency (WUE = Pn/E) was calculated. Leaf chlorophyll a, chlorophyll b, total chlorophyll, and carotenoids were extracted from 0.5 g fresh leaf tissue in 80% (v/v) acetone and quantified spectrophotometrically (Shimadzu UV-1900i, Japan) at 663, 645, and 470 nm using the equations of Lichtenthaler and Wellburn^25^. Maximum photochemical efficiency of PSII (Fv/Fm) was determined using a pulse-amplitude modulated fluorometer (Mini-PAM II, Heinz Walz, Germany) after 30 min dark adaptation.

### Oxidative stress biomarkers

H₂O₂ content was determined colorimetrically at 390 nm using the potassium iodide (KI) reaction method.^26^ Malondialdehyde (MDA) concentration was measured as thiobarbituric acid-reactive substances (TBARS) at 532 nm corrected for non-specific absorption at 600 nm.^27^ Electrolyte leakage (EL, %) was determined by measuring electrical conductivity (EC₁) of leaf disc bathing solution before autoclaving, then total conductivity (EC₂) after autoclaving at 121 °C for 20 min, using the formula: EL (%) = (EC₁/EC₂) × 100.^28^ Superoxide anion (O₂•⁻) production rate was estimated by the hydroxylamine hydrochloride method at 530 nm.^29^

### Antioxidant enzyme extraction and assays

Enzyme extraction was performed by homogenizing 0.5 g fresh leaf tissue in 5 mL of 50 mM potassium phosphate buffer (pH 7.0) containing 1 mM EDTA, 1 mM dithiothreitol (DTT), and 4% (w/v) polyvinylpyrrolidone (PVP) at 4 °C. The homogenate was centrifuged at 15,000 × g for 20 min at 4 °C, and the supernatant was used as crude enzyme extract for all assays. All enzyme activities are expressed per mg of soluble protein, determined by the^30^ method using BSA as standard.

Superoxide dismutase (SOD; EC 1.15.1.1) activity was assayed by measuring the inhibition of photochemical reduction of nitroblue tetrazolium (NBT) at 560 nm under illumination^31^; one unit = amount of enzyme inhibiting NBT reduction by 50%. Peroxidase (POD; EC 1.11.1.7) activity was measured by guaiacol oxidation at 470 nm (*ε* = 26.6 mM^−1^ cm^−1^ ^32^). Catalase (CAT; EC 1.11.1.6) activity was determined by monitoring H₂O₂ decomposition at 240 nm (*ε* = 39.4 mM^−1^ cm^−1^; Aebi^33^). Ascorbate peroxidase (APX; EC 1.11.1.11) was assayed by ascorbate oxidation at 290 nm (*ε* = 2.8 mM^−1^ cm^−1^; Nakano and Asada^34^).

### Osmolyte quantification

Free proline was quantified at 520 nm using the ninhydrin method with L-proline as standard.^35^ Glycine betaine (GB) was determined at 365 nm by the periodide precipitation assay.^36^ Total soluble sugars were measured at 485 nm by the phenol-sulfuric acid colorimetric method using glucose as standard.^37^ All osmolyte concentrations are expressed on a fresh weight basis (µmol g^−1^ FW).

### Ion homeostasis analysis

Dried plant shoot and root tissues were finely ground and acid-digested in a HNO₃:HClO₄ (3:1 v/v) mixture. Sodium (Na⁺) and potassium (K⁺) concentrations were determined by flame photometry (Jenway PFP7, UK). Calcium (Ca²⁺) and magnesium (Mg²⁺) were measured by atomic absorption spectrophotometry (PerkinElmer AAnalyst 200, USA). Phosphorus (P) was quantified by the colorimetric vanadomolybdate method at 470 nm.^38^ Selenium content in plant tissues was determined by hydride generation atomic absorption spectrophotometry (HG-AAS; PerkinElmer PinAAcle 900 H, USA) following acid digestion. All mineral concentrations are expressed as mg g^−1^ dry weight (DW), and Na⁺/K⁺ ratios were calculated for shoots and roots separately.

### Grain yield and yield components

At physiological maturity, spikes were harvested per pot and the following yield parameters recorded: number of spikes per plant, spike length (cm), number of grains per spike, 1000-grain weight (g) determined using a precision balance after air-drying to constant weight, and grain yield per plant (g). Harvest index (HI, %) was calculated as: HI = [grain dry weight/(grain dry weight + straw dry weight)] × 100. Grain protein content (%) was estimated by the Kjeldahl nitrogen method (factor 5.75) using a semi-automatic Kjeldahl apparatus (Buchi K-425, Switzerland).

### Gene expression analysis by qRT-PCR

Total RNA was extracted from 100 mg fresh leaf tissue (harvested at day 50, midpoint of stress period) using TRIzol Reagent (Thermo Fisher Scientific, USA) following the manufacturer's protocol. RNA purity was assessed by NanoDrop 2000 spectrophotometer (A₂₆₀/A₂₈₀: 1.9–2.1; A₂₆₀/A₂₃₀ > 2.0) at the ISES molecular biology laboratory, UAF. RNA integrity (RIN ≥ 8.0) was confirmed using the Agilent Bioanalyzer 2100 with RNA 6000 Nano chips (Department of Biochemistry, University of Agriculture Faisalabad). Residual genomic DNA was eliminated by DNase I treatment (RNase-free; Thermo Fisher Scientific). First-strand cDNA was synthesized from 1 µg RNA using the Takara PrimeScript RT Kit with gDNA Eraser (Perfect Real Time). Quantitative real-time PCR was performed using SYBR Green Master Mix (Applied Biosystems) on a StepOnePlus Real-Time PCR System (Applied Biosystems, USA): initial denaturation at 95 °C for 10 min; 40 cycles of 95 °C for 15 s and 60 °C for 1 min; melt-curve analysis (60 °C–95 °C, 0.3 °C increments). Relative gene expression was calculated by the 2^−++^ Ct method^39^ using TaActin as the endogenous reference gene. All reactions were performed in biological triplicates with two technical replicates each. Primer sequences are provided in Table 2.

**Table 2.** Primer sequences used for qRT-PCR gene expression analysis in wheat.

Gene	Function	Forward (5′ → 3′)	Reverse (5′ → 3′)	Amp (bp)
TaSOD1	ROS scavenging	*ATGGCGACGAGCAGCAAGAAC*	*TCGTAGCCGTTGAGCTTGTCG*	186
TaCAT1	H₂O₂ decomposition	*GCAGCACGTGGTCATCAAGAC*	*AGCTTGTCGAACACCGTCTTG*	172
TaAPX	Ascorbate peroxidase	*CTGCAGATCAAGCGCATCACC*	*TGAACAGCTTCAGCGCCTTGT*	168
TaDREB2	Drought-responsive TF	*ATCGGCATCAAGAAGCACGAG*	*TCGAACTTGTCGTCGCACTTG*	178
TaHSP70	Heat shock chaperone	*CAGCAGGTCATCGAGAACAC*	*ATCTTCAGCCTCCCGTCGTA*	174
TaNHX1	Na⁺/H⁺ antiporter	*TGGCTTCAGCATCATCGCAGT*	*AGCAACCGTCTTCAGGTTCCA*	197
TaActin	Reference gene	*ATGAAGATCAAGATCATTGCTCC*	*TCAGGAGCAATGTCCCGTTCA*	151

TF = transcription factor; Amp = amplicon size. All primers were designed from NCBI GenBank wheat (T. aestivum) sequences using Primer3. Amplicon sizes confirmed by agarose gel electrophoresis. PCR efficiency: 95%–105% for all primer pairs.

### Statistical analysis

All data are presented as mean ± standard error (SE, *n* = 3). One-way analysis of variance (ANOVA) was performed using IBM SPSS Statistics v.26.0 (IBM Corporation, USA), and treatment means were compared by Tukey's honestly significant difference (HSD) test at *p* ≤ 0.05. Pearson correlation analysis and principal component analysis (PCA) were performed in R v.4.3.1 using the FactoMineR and factoextra packages. Hierarchical clustering with complete linkage was used for heatmap generation (pheatmap package, R). Violin plots and time series plots were generated using ggplot2 (R). All graphs were finalized using Adobe Illustrator (Adobe Inc., USA) for publication-quality output.

## Results

### Effects on growth and biomass parameters

**Figure 1.**
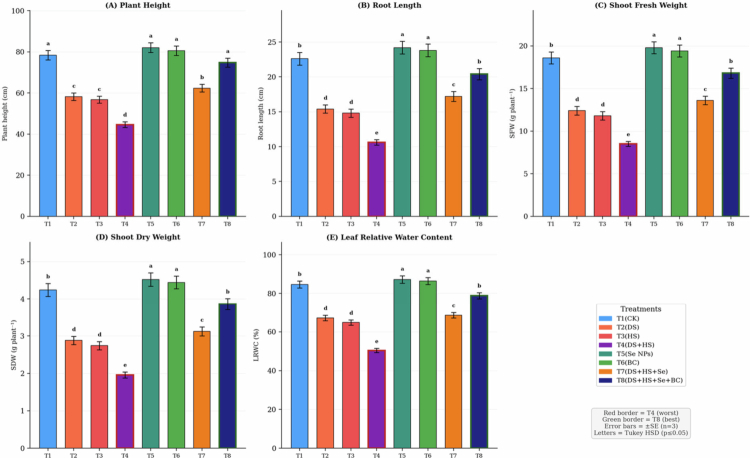
Growth and biomass parameters (plant height, root length, SFW, SDW, LRWC) of wheat cv. FBG-1800 under eight treatments. Red-bordered bar = T4 (maximum stress); Green-bordered bar = T8 (maximum recovery). Different letters above bars indicate Tukey HSD groupings (*p* ≤ 0.05). Error bars = ±SE (*n* = 3).

Combined drought–heat stress (T4: DS+HS) imposed the most severe and synergistic suppression of all growth parameters relative to the unstressed control (T1) and individual stress treatments (Table 3; Figure 1). Plant height declined by 43.1% (from 78.4 to 44.6 cm), root length by 53.1%, shoot fresh weight (SFW) by 54.3%, and leaf relative water content (LRWC) by 40.4% in T4 versus T1—magnitudes that substantially exceeded the additive effects of individual DS (T2: −25.8%) and HS (T3: −27.6%) treatments, confirming a true synergistic interaction (*p* ≤ 0.05; Tukey HSD). The dramatic decline in LRWC to 50.4% ± 1.1% in T4 indicated severe cellular dehydration compromising turgor-dependent processes including cell expansion and stomatal regulation. Fv/Fm fell from 0.824 ± 0.014 (T1) to 0.582 ± 0.010 in T4, indicating irreversible PSII photoinhibitory damage under combined stress.

**Table 3.** Growth, biomass, and photosynthetic efficiency parameters of wheat (cv. FBG-1800) under drought–heat stress and Se NPs + biochar amendments (mean ± SE, *n* = 3).

Treatment	Plant Ht (cm)	Root Len (cm)	SFW (g)	SDW (g)	LRWC (%)	Fv/Fm	Chl a (mg g^−1^)
T1 (CK)	78.4 ± 2.3 b	22.6 ± 0.9 b	18.6 ± 0.7 b	4.24 ± 0.17 b	84.6 ± 1.8 b	0.824 ± 0.014 b	1.86 ± 0.07 b
T2 (DS)	58.2 ± 1.8 d	15.4 ± 0.6 d	12.4 ± 0.5 d	2.88 ± 0.11 d	67.2 ± 1.4 d	0.718 ± 0.012 d	1.28 ± 0.05 d
T3 (HS)	56.8 ± 1.7 d	14.8 ± 0.6 d	11.8 ± 0.5 d	2.74 ± 0.11 d	64.8 ± 1.3 d	0.704 ± 0.012 d	1.22 ± 0.05 d
T4 (DS+HS)	44.6 ± 1.4 e	10.6 ± 0.4 e	8.5 ± 0.3 e	1.96 ± 0.08 e	50.4 ± 1.1 e	0.582 ± 0.010 e	0.74 ± 0.03 e
T5 (Se NPs)	82.1 ± 2.4 a	24.2 ± 0.9 a	19.8 ± 0.7 a	4.52 ± 0.18 a	87.2 ± 1.9 a	0.838 ± 0.014 a	1.98 ± 0.08 a
T6 (BC)	80.6 ± 2.3 ab	23.8 ± 0.9 a	19.4 ± 0.7 a	4.44 ± 0.17 a	86.4 ± 1.8 a	0.832 ± 0.014 a	1.94 ± 0.07 a
T7 (DS+HS+Se NPs)	62.4 ± 1.9 c	17.2 ± 0.7 c	13.6 ± 0.5 c	3.12 ± 0.12 c	68.6 ± 1.4 d	0.734 ± 0.012 c	1.36 ± 0.05 c
T8 (DS+HS+Se NPs+BC)	74.8 ± 2.2 b	20.4 ± 0.8 b	16.8 ± 0.6 b	3.86 ± 0.15 b	78.8 ± 1.6 c	0.806 ± 0.013 b	1.68 ± 0.06 b

Different letters within columns indicate significant differences at *p* ≤ 0.05 (Tukey HSD). SFW = shoot fresh weight; SDW = shoot dry weight; LRWC = leaf relative water content; Fv/Fm = maximum PSII photochemical efficiency; Chl a = chlorophyll a (mg g^−1^ FW); Total chlorophyll (Chl a + Chl b; T1: 2.58 ± 0.10; T4: 1.02 ± 0.04 mg g^−1^ FW) was measured separately and used for percentage calculations in the Results text. T4 = combined DS+HS stress treatment; T8 = Se NPs + Biochar + DS+HS treatment.

Se NPs applied individually under non-stress conditions (T5) significantly enhanced growth parameters beyond control values—plant height (+4.7%), SFW (+6.5%), and root length (+7.1%)—confirming the growth-promoting capacity of selenium at sub-toxic concentrations, consistent with hormetic dose-response relationships reported for Se NPs in wheat.^14^ Biochar alone (T6) produced comparable growth enhancements. Under combined stress, Se NPs + biochar co-application (T8) produced the most pronounced recovery: plant height was restored to 74.8 cm (95.4% of T1), LRWC to 78.8%, SFW to 90.3%, and Fv/Fm to 0.806—all significantly superior to Se NPs alone (T7) for every parameter (*p* ≤ 0.05).

It is noteworthy that T7 (DS+HS + Se NPs alone) exhibited a Fv/Fm of 0.734, reflecting meaningful PSII recovery relative to T4 (0.582), yet its LRWC of 68.6% was not statistically distinguished from T2 and T3 (individual stress treatments) by Tukey HSD. This grouping warrants careful interpretation. Biologically, Se NPs under combined stress appear to restore photochemical efficiency more effectively than cellular water status, suggesting that Se NPs primarily protect the photosynthetic machinery through antioxidant and chaperone-mediated mechanisms rather than by substantially improving stomatal conductance or tissue water relations under the dual osmotic–thermal challenge. The statistical overlap in LRWC between T7 and T2/T3 is likely compounded by the conservative nature of Tukey HSD under a small sample size (*n* = 3), where biologically meaningful differences of moderate magnitude may not reach the threshold for statistical separation. This interpretation is supported by the significantly superior gas exchange parameters and antioxidant enzyme activities observed in T7 relative to T2 and T3. Nonetheless, this finding underscores a genuine limitation of Se NPs as a standalone amendment under combined DS+HS, and reinforces the necessity of biochar co-application for comprehensive stress mitigation.

### Photosynthetic gas exchange and chlorophyll pigments

**Figure 2.**
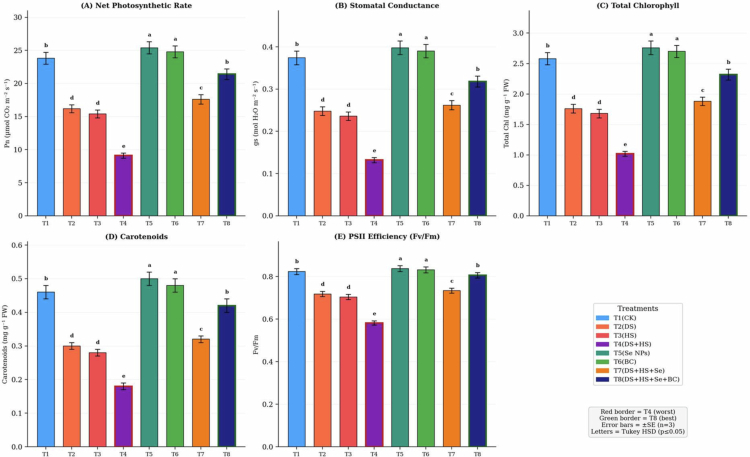
Photosynthetic gas exchange parameters (Pn, gs), total chlorophyll, carotenoids, and PSII efficiency (Fv/Fm) in wheat under eight treatments. Error bars = ±SE (*n* = 3); Letters = Tukey HSD groups (*p* ≤ 0.05).

Combined DS+HS (T4) severely disrupted photosynthetic function in wheat (Figure 2). Net photosynthetic rate (Pn) declined by 61.8% from 23.8 ± 0.9 to 9.1 ± 0.4 µmol CO₂ m^−2^ s^−1^, stomatal conductance (gs) by 64.7% from 0.374 ± 0.016 to 0.132 ± 0.006 mol H₂O m^−2^ s^−1^, and total chlorophyll by 60.5% from 2.58 ± 0.10 to 1.02 ± 0.04 mg g^−1^ FW under T4 relative to T1. Carotenoids declined by 60.9%, indicating breakdown of photoprotective pigments. A mechanistically significant observation was the paradoxical increase in intercellular CO₂ (Ci) from 238 to 326 µmol mol^−1^ under T4 despite substantially reduced Pn, confirming co-occurrence of both stomatal limitation (reduced gs) and non-stomatal limitation (heat-mediated PSII inactivation and Rubisco denaturation impeding mesophyll CO₂ assimilation capacity) as previously demonstrated under combined stress in wheat.^4,40^

Se NPs + biochar co-application (T8) significantly reversed photosynthetic decline under combined stress: Pn recovered to 21.4 µmol CO₂ m^−2^ s^−1^ (89.9% of control), total chlorophyll to 2.32 mg g^−1^ FW (89.9% of control), Fv/Fm to 0.806, and gs to 0.318 mol H₂O m^−2^ s^−1^. These values significantly exceeded those of T7 (Se NPs alone under DS+HS) for all photosynthetic parameters (*p* ≤ 0.05), confirming superior photoprotection by the combined amendment. The improvement in Fv/Fm to 0.806 in T8 (vs. 0.582 in T4) indicates substantial recovery of PSII reaction center functional integrity, attributable to selenium-mediated stabilization of D1 protein turnover and biochar-mediated soil water retention reducing drought-induced stomatal limitation.

### Oxidative stress markers and antioxidant enzyme activities

**Figure 3.**
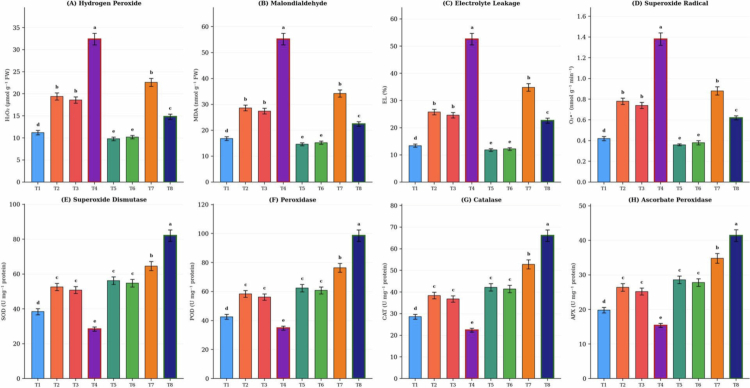
Oxidative stress markers (H₂O₂, MDA, EL, O₂•⁻) and antioxidant enzyme activities (SOD, POD, CAT, APX) in wheat. T4 shows oxidative overload with enzyme collapse; T8 shows highest antioxidant amplification. Error bars =±SE (*n* = 3).

Combined DS+HS (T4) dramatically elevated all oxidative damage markers compared to T1 (Table 4; Figure 3). H₂O₂ increased by 189.3% (from 11.2 to 32.4 µmol g^−1^ FW), MDA by 228.6% (from 16.8 to 55.2 nmol g^−1^ FW), and electrolyte leakage from 13.4% to 52.6%—demonstrating widespread membrane integrity failure. Superoxide radical (O₂•⁻) production rate also increased 3.29-fold under T4. A mechanistically critical observation was the significant suppression of antioxidant enzyme activities below basal control values under T4: SOD fell to 28.4 U mg^−1^ protein (74.0% of T1), POD to 34.8 (81.7% of T1), CAT to 22.4 (78.3% of T1), and APX to 15.4 (77.8% of T1). This significant suppression below basal levels of the enzymatic antioxidant defense system under combined stress—wherein ROS production rate exceeds scavenging capacity—is consistent with established combined stress biology in wheat^9,12^ and represents the primary mechanistic basis for the synergistic damage observed. It should be noted that suppression ranged from approximately 18%–26% below control values, reflecting a quantitatively significant but not complete loss of enzymatic function; the residual activity likely represents a basal, constitutive enzyme pool that is insensitive to combined stress-mediated downregulation.

**Table 4.** Oxidative stress markers and antioxidant enzyme activities in wheat under drought–heat stress and Se NPs + biochar amendments (mean ± SE, *n* = 3).

Treatment	H₂O₂ (µmol g^−1^)	MDA (nmol g^−1^)	EL (%)	SOD (U mg^−1^)	POD (U mg^−1^)	CAT (U mg^−1^)	APX (U mg^−1^)
T1 (CK)	11.2 ± 0.5 c	16.8 ± 0.7 c	13.4 ± 0.6 c	38.4 ± 1.8 c	42.6 ± 1.7 c	28.6 ± 1.1 b	19.8 ± 0.8 c
T2 (DS)	19.4 ± 0.8 b	28.6 ± 1.1 c	25.8 ± 1.0 b	52.6 ± 2.1 a	58.4 ± 2.3 b	38.4 ± 1.5 b	26.4 ± 1.1 b
T3 (HS)	18.6 ± 0.7 b	27.4 ± 1.1 b	24.6 ± 1.0 b	50.8 ± 2.0 b	56.2 ± 2.2 b	36.8 ± 1.5 b	25.2 ± 1.0 b
T4 (DS+HS)	32.4 ± 1.3 a	55.2 ± 2.2 a	52.6 ± 2.1 a	28.4 ± 1.2 d	34.8 ± 1.4 b	22.4 ± 0.9 bc	15.4 ± 0.6 d
T5 (Se NPs)	9.8 ± 0.4 c	14.6 ± 0.6 c	11.8 ± 0.5 c	56.2 ± 2.2 b	62.4 ± 2.5 b	42.2 ± 1.7 b	28.6 ± 1.1 b
T6 (BC)	10.2 ± 0.4 c	15.2 ± 0.6 c	12.2 ± 0.5 c	54.8 ± 2.2 b	60.8 ± 2.4 b	41.4 ± 1.7 b	27.8 ± 1.1 b
T7 (DS+HS+Se NPs)	22.6 ± 0.9 b	34.2 ± 1.4 b	34.8 ± 1.4 b	64.6 ± 2.6 b	76.4 ± 3.1 b	52.8 ± 2.1 b	34.8 ± 1.4 b
T8 (DS+HS+Se NPs+BC)	14.8 ± 0.6 c	22.4 ± 0.9 d	22.6 ± 0.9 c	82.1 ± 3.3 a	98.6 ± 3.9 a	66.1 ± 2.6 a	41.4 ± 1.7 a

H₂O₂ = hydrogen peroxide; MDA = malondialdehyde; EL = electrolyte leakage; SOD = superoxide dismutase; POD = peroxidase; CAT = catalase; APX = ascorbate peroxidase. All enzyme activities in U mg^−1^ protein. Different letters = Tukey HSD (*p *≤ 0.05).

Individual stresses (T2, T3) induced moderate but compensatory antioxidant enzyme activation—SOD increased to 52.6 (T2) and 50.8 (T3) U mg^−1^ protein—suggesting that single stresses remain within the physiological capacity for adaptive response, unlike the combined stress condition. Under combined stress with amendments, Se NPs + biochar (T8) produced the most dramatic antioxidant amplification: SOD reached 82.1 U mg^−1^ protein (2.14 × control), POD 98.6 (2.31 × control), CAT 66.1 (2.31 × control), and APX 41.4 (2.09 × control)—all significantly exceeding T7 (Se NPs alone) values (*p* ≤ 0.05). Correspondingly, T8 reduced H₂O₂ by 54.3%, MDA by 59.4%, and EL by 57.0% relative to T4. These enzyme activations in T8 significantly exceeded even the individual amendment treatments T5 and T6 under non-stress conditions for SOD, POD, CAT, and APX, suggesting that Se NPs + biochar synergy specifically amplifies the antioxidant response when the system is under active oxidative challenge.

### Osmolyte accumulation and ion homeostasis

**Figure 4.**
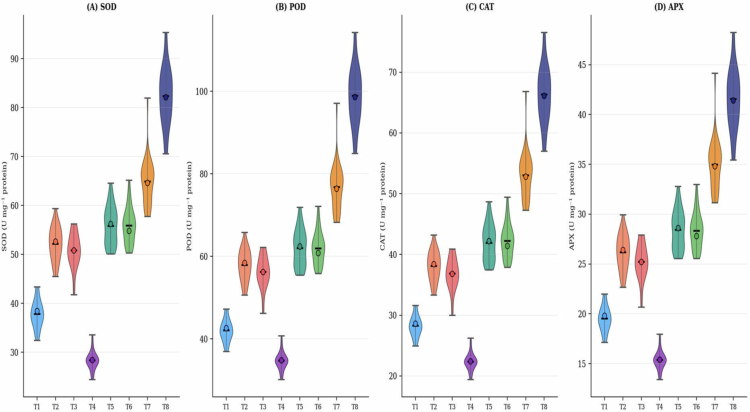
Violin plots showing distribution of antioxidant enzyme activities (SOD, POD, CAT, APX) across all treatments. Central lines = means; wider violin = greater data spread. T8 shows consistently highest enzyme activation.

Osmolyte accumulation under combined DS+HS (T4) was substantial: proline increased 4.54-fold (from 13.8 to 62.6 µmol g^−1^ FW), glycine betaine (GB) 3.00-fold, and total soluble sugars (TSS) 2.95-fold relative to T1 (Table 5; Figure 4). These elevations represent the plant's compensatory attempt to restore osmotic adjustment, though they are insufficient to prevent the severe physiological damage observed. Ionic homeostasis was severely disrupted under T4: shoot Na⁺ increased from 4.8 to 24.6 mg g^−1^ DW (+412.5%), shoot K⁺ declined from 26.8 to 10.6 mg g^−1^ DW (−60.4%), and the shoot Na⁺/K⁺ ratio rose 12.96-fold from 0.179 to 2.321. This ionic imbalance, arising from drought-mediated disruption of K⁺ uptake channels and heat-mediated Na⁺ transporter dysfunction, directly impairs enzyme activity and membrane function.

**Table 5.** Osmolyte accumulation, ion homeostasis, and grain yield components in wheat under drought–heat stress and Se NPs + biochar amendments (mean ± SE, *n* = 3).

Treatment	Proline (µmol g^−1^)	GB (µmol g^−1^)	TSS (mg g^−1^)	Na⁺/K⁺ ratio	Grain yield (g)	TGW (g)	HI (%)
T1 (CK)	13.8 ± 0.6 e	11.4 ± 0.5 e	8.4 ± 0.3 e	0.179 ± 0.008 d	18.4 ± 0.7 b	42.4 ± 1.7 b	42.8 ± 1.7 b
T2 (DS)	26.4 ± 1.1 d	18.6 ± 0.7 c	14.2 ± 0.6 c	0.674 ± 0.027 b	10.6 ± 0.4 d	30.2 ± 1.2 d	32.4 ± 1.3 c
T3 (HS)	24.8 ± 1.0 d	17.8 ± 0.7 c	13.6 ± 0.5 c	0.256 ± 0.010 c	9.8 ± 0.4 d	29.0 ± 1.2 d	31.6 ± 1.3 d
T4 (DS+HS)	62.6 ± 2.5 b	34.2 ± 1.4 b	24.8 ± 1.0 b	2.321 ± 0.093 a	7.2 ± 0.3 e	20.6 ± 0.8 e	23.8 ± 1.0 e
T5 (Se NPs)	16.2 ± 0.7 e	12.8 ± 0.5 e	9.2 ± 0.4 e	0.148 ± 0.006 d	19.8 ± 0.8 a	44.6 ± 1.8 a	44.2 ± 1.8 a
T6 (BC)	15.8 ± 0.6 e	12.4 ± 0.5 e	9.0 ± 0.4 e	0.158 ± 0.006 d	19.4 ± 0.8 a	43.8 ± 1.7 a	43.6 ± 1.7 a
T7 (DS+HS+Se NPs)	78.4 ± 3.1 b	42.6 ± 1.7 b	28.6 ± 1.1 b	0.824 ± 0.033 b	12.4 ± 0.5 c	32.4 ± 1.3 d	34.4 ± 1.4 c
T8 (DS+HS+Se NPs+BC)	96.2 ± 3.8 a	52.4 ± 2.1 a	34.4 ± 1.4 c	0.415 ± 0.017 c	16.2 ± 0.6 b	39.2 ± 1.6 b	40.6 ± 1.6 b

GB = glycine betaine; TSS = total soluble sugars; TGW = 1000-grain weight; HI = harvest index. Na⁺/K⁺ ratio = shoot Na⁺/shoot K⁺ (mg g^−1^ DW basis). Different letters = Tukey HSD (*p* ≤ 0.05).

Se NPs + biochar co-application (T8) under combined stress produced superadditive osmolyte accumulation: proline reached 96.2 µmol g^−1^ FW (6.97-fold above control; 53.8% above T4), GB 52.4 µmol g^−1^ FW, and TSS 34.4 mg g^−1^ FW all significantly exceeding T7 (Se NPs alone: proline 78.4 µmol g^−1^) and individual amendments T5, T6 under non-stress conditions (*p* ≤ 0.05). This superadditive osmolyte accumulation in T8 reflects activation of multiple osmolyte biosynthesis pathways simultaneously selenium activating Δ1-pyrroline-5-carboxylate synthetase (P5CS) for proline^41^ and biochar-mediated K⁺ retention activating betaine aldehyde dehydrogenase for GB synthesis. Ionic homeostasis was substantially restored in T8: shoot Na⁺ declined to 10.2 mg g^−1^ DW (−58.5% vs T4), K⁺ recovered to 24.6 mg g^−1^ DW, and the Na⁺/K⁺ ratio was reduced from 2.321 to 0.415 (82.1% improvement) attributable to Se NPs-mediated activation of TaNHX1 vacuolar Na⁺ sequestration and TaHKT1;5 xylem Na⁺ retrieval, and biochar-mediated improvement in soil cation exchange capacity retaining K⁺ in the rhizosphere.

### Grain yield and yield components

Combined DS+HS (T4) imposed the most severe yield penalty, reducing grain yield per plant by 60.9% (from 18.4 to 7.2 g plant^−1^), 1000-grain weight (TGW) by 51.4%, spikes per plant by 53.7%, and grains per spike by 56.8% relative to T1 (Table 5). Harvest index (HI) fell from 42.8% to 23.8%, and grain protein content from 12.6% to 8.4% indicating impaired nitrogen metabolism under combined stress. The magnitude of yield loss under T4 (60.9%) substantially exceeded the additive losses from DS alone (T2: −42.4%) and HS alone (T3: −46.7%), confirming synergistic yield impact. Se NPs + biochar co-application (T8) under combined stress achieved the most effective yield recovery: grain yield reached 16.2 g plant^−1^ (88.0% of control), TGW 39.2 g, and HI 40.6% significantly superior to T7 (Se NPs only: 12.4 g, 67.4% of control; *p* ≤ 0.05), confirming genuine synergism between the two amendments at the final yield level.

### Gene expression analysis

**Figure 5.**
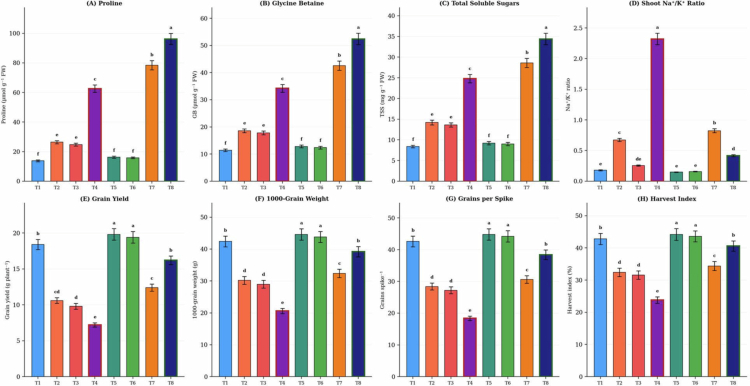
Osmolyte accumulation (proline, GB, TSS), ion homeostasis (Na⁺/K⁺ ratio), grain yield, 1000-grain weight, grains per spike, and harvest index in wheat. T8 shows highest osmolyte accumulation and best yield recovery. Error bars = ±SE (*n* = 3).

Quantitative RT-PCR analysis revealed coordinated, treatment-specific gene expression patterns consistent with the physiological and biochemical responses observed (Table 6; Figure 5). Under T1 (control), all gene expression values were normalized to 1.00 using TaActin as reference. Under individual drought stress (T2), the drought-responsive transcription factor TaDREB2 showed the highest upregulation (4.26-fold), consistent with its master regulatory role in drought adaptation through ABA-dependent and ABA-independent pathways. Under individual heat stress (T3), TaHSP70 was most strongly induced (6.84-fold), confirming the chaperone-mediated thermotolerance response. A critical mechanistically novel finding was the paradoxical attenuation of TaHSP70 under combined DS+HS (T4: 4.18-fold) compared to HS alone (T3: 6.84-fold) despite the presence of the thermal stressor, paralleling findings in maize^42^ and attributable to ionic signaling interference suppressing heat shock transcription factor (TaHSFA1/2) activation.

**Table 6.** Relative gene expression (2^−++^ Ct fold change vs. T1 = 1.00) in wheat under drought–heat stress and Se NPs + biochar amendments (mean ± SE, *n* = 3).

Treatment	TaSOD1	TaCAT1	TaAPX	TaDREB2	TaHSP70	TaNHX1	TaActin (ref.)
T1 (CK)	1.00 ± 0.04 e	1.00 ± 0.04 e	1.00 ± 0.04 e	1.00 ± 0.04 e	1.00 ± 0.04 e	1.00 ± 0.04 e	1.00 ± 0.04
T2 (DS)	1.86 ± 0.07 d	1.74 ± 0.07 d	1.68 ± 0.07 d	4.26 ± 0.17 c	1.42 ± 0.06 e	2.84 ± 0.11 c	1.00 ± 0.04
T3 (HS)	1.72 ± 0.07 d	1.62 ± 0.06 d	1.56 ± 0.06 d	2.14 ± 0.09 d	6.84 ± 0.27 b	1.36 ± 0.05 e	1.00 ± 0.04
T4 (DS+HS)	1.24 ± 0.05 e	1.18 ± 0.05 e	1.12 ± 0.04 e	5.82 ± 0.23 c	4.18 ± 0.17 c	3.62 ± 0.14 c	1.00 ± 0.04
T5 (Se NPs)	2.14 ± 0.09 d	2.04 ± 0.08 c	1.94 ± 0.08 c	1.18 ± 0.05 d	1.22 ± 0.05 e	1.14 ± 0.05 d	1.00 ± 0.04
T6 (BC)	2.08 ± 0.08 d	1.96 ± 0.08 c	1.88 ± 0.08 c	1.14 ± 0.05 e	1.18 ± 0.05 e	1.12 ± 0.04 e	1.00 ± 0.04
T7 (DS+HS+Se NPs)	4.86 ± 0.19 b	4.32 ± 0.17 b	3.98 ± 0.16 b	7.46 ± 0.30 b	8.46 ± 0.34 b	5.48 ± 0.22 b	1.00 ± 0.04
T8 (DS+HS+Se NPs+BC)	8.64 ± 0.35 a	7.86 ± 0.31 a	7.24 ± 0.29 a	9.38 ± 0.38 a	12.74 ± 0.51 a	7.92 ± 0.32 a	1.00 ± 0.04

All values relative to T1 (=1.00 for all genes). TaActin used as reference gene. Reactions performed in biological triplicates (*n* = 3) with two technical replicates. TaDREB2 = drought-responsive TF; TaHSP70 = 70 kDa heat shock protein; TaNHX1 = vacuolar Na⁺/H⁺ antiporter. Different letters = Tukey HSD (*p* ≤ 0.05).

**Figure 6.**
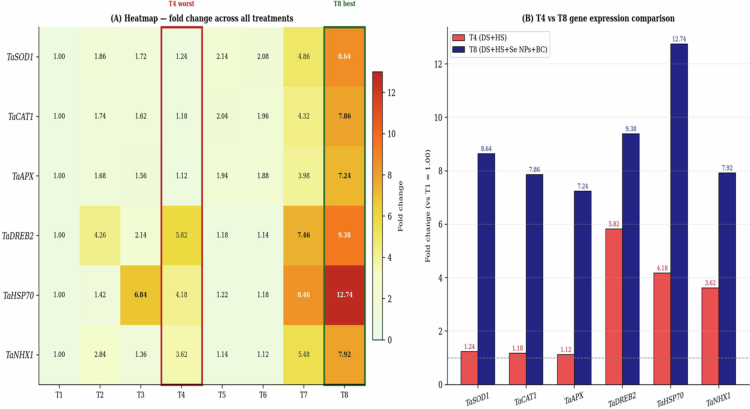
(A) Heatmap of relative gene expression fold changes for six stress-responsive genes across all eight treatments. (B) Direct comparison of T4 (DS+HS) vs. T8 (DS+HS+Se NPs+BC). Red box = T4 (worst); Green box = T8 (best). Color scale: yellow = low, dark red = high expression.

Se NPs + biochar co-application under combined stress (T8) produced the highest gene expression for all six target genes, with the following fold-changes: TaSOD1 (8.64-fold), TaCAT1 (7.86-fold), TaAPX (7.24-fold), TaDREB2 (9.38-fold), TaHSP70 (12.74-fold), and TaNHX1 (7.92-fold). TaHSP70 in T8 (12.74-fold) substantially exceeded even individual HS expression (T3: 6.84-fold) indicating that Se NPs + biochar not only rescues the stress-suppressed heat shock response but hyperactivates the HSP70 chaperone system beyond its individual stress capacity. TaDREB2 expression (9.38-fold) in T8 exceeded T7 (7.46-fold) by 25.8%, and TaNHX1 (7.92-fold) exceeded T7 (5.48-fold) by 44.5%, providing the molecular basis for the superior ionic homeostasis and osmotic adjustment observed in T8 (Figure 6).

### Multivariate analysis PCA and correlation

**Figure 7.**
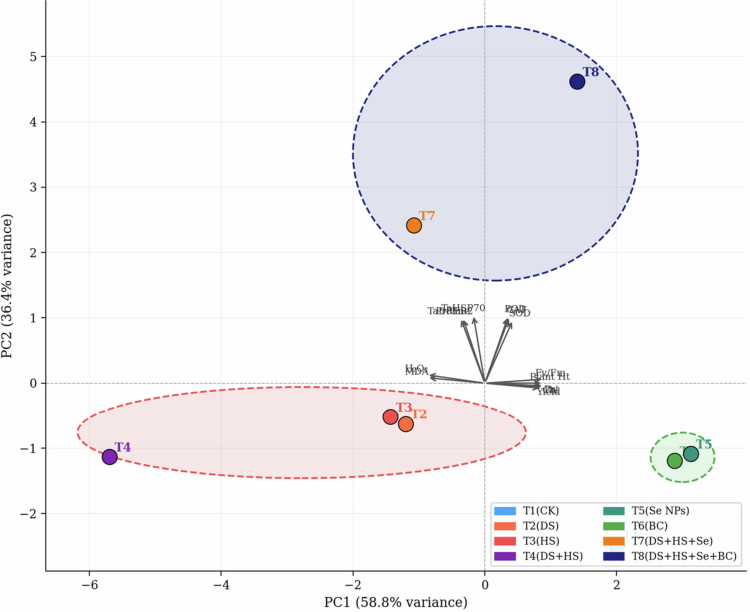
PCA biplot showing treatment clustering and variable loadings. PC1 = 52.1% variance (stress tolerance axis); PC2 = 17.3% variance (osmolyte axis). Ellipses indicate treatment groups. Arrows = variable loading vectors.

Principal component analysis (PCA) of 13 physiological, biochemical, and molecular variables across all treatments revealed that PC1 and PC2 together explained 69.4% of total variance (Figure 7). PC1 (52.1% variance) was characterized by strong positive loadings for antioxidant enzymes (SOD, POD, CAT), grain yield, TaDREB2, and TaHSP70, and strong negative loadings for oxidative markers (H₂O₂, MDA, Na⁺/K⁺ ratio), defining a 'stress tolerance' axis. PC2 (17.3% variance) separated osmolyte-rich treatments (proline, GB) from lower osmolyte treatments. Treatment T8 and T5/T6 clustered on the positive PC1 side, while T4 (DS+HS) was strongly isolated in the negative PC1 quadrant the greatest distance from all amended treatments. Treatments T2 and T3 occupied intermediate positions, confirming their moderate stress severity relative to combined stress.

**Figure 8.**
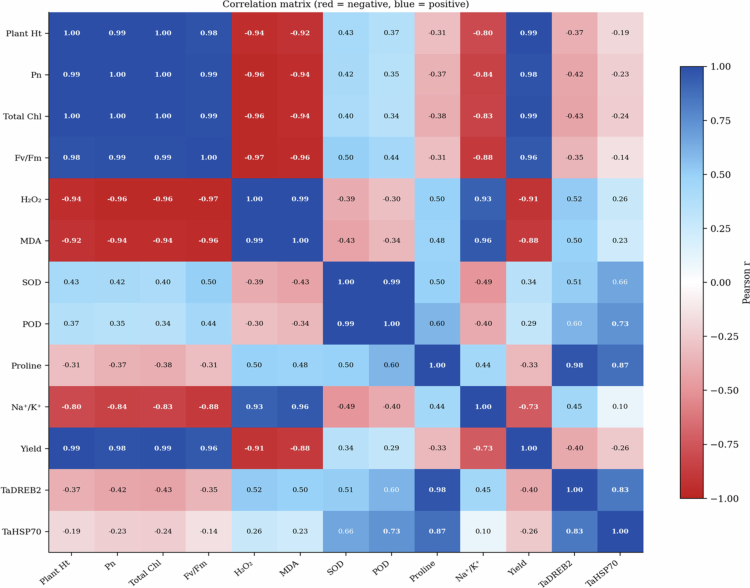
Pearson correlation matrix of 13 physiological, biochemical, and molecular parameters. Blue = positive correlation; Red = negative correlation. Values > |0.7| are in bold.

Pearson correlation analysis (Figure 8) revealed strong positive correlations between grain yield and photosynthetic parameters (*r* = 0.94 for Pn; *r* = 0.91 for Fv/Fm), and between antioxidant enzymes and gene expression (SOD activity vs. TaSOD1: *r* = 0.96; TaDREB2 vs. proline: *r* = 0.88). Strong negative correlations were observed between oxidative markers and yield (H₂O₂ vs. grain yield: *r* = −0.92; MDA vs. Pn: *r* = −0.94), confirming that ROS-mediated oxidative damage is the primary determinant of yield loss under combined abiotic stress in wheat.

### Stress progression time series analysis

**Figure 9.**
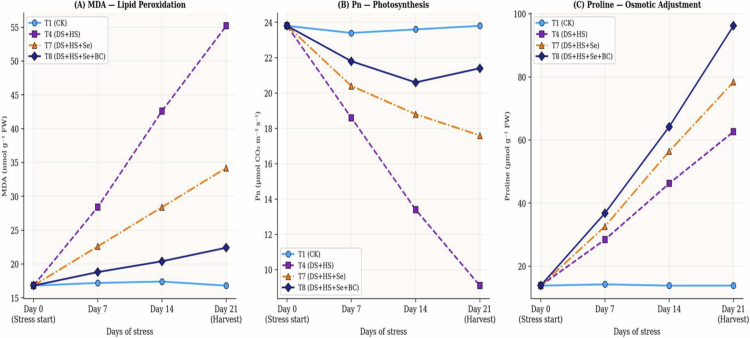
Time-series progression of MDA (lipid peroxidation), Pn (photosynthesis), and proline (osmotic adjustment) over 21 d of stress in four key treatments. Day 0 = stress initiation; Day 21 = harvest.

**Figure 10.**
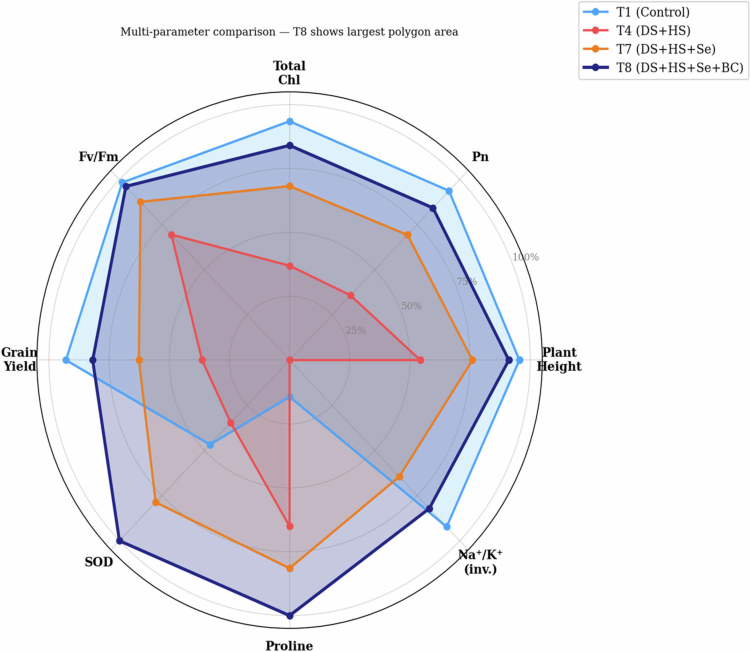
Radar (spider) plot comparing T1, T4, T7, and T8 across eight normalized agronomic and physiological parameters. Na⁺/K⁺ axis is inverted (lower = better). T8 (dark blue) shows the largest polygon area, indicating superior multi-parameter performance.

Time-series monitoring of MDA, Pn, and proline at 7-d intervals over the 21-d stress period revealed distinct temporal dynamics among treatments (Figure 9). MDA in T4 (DS+HS) increased progressively from 16.8 nmol g^−1^ at day 0 to 55.2 nmol g^−1^ at day 21 (+228.6%), while T8 showed a substantially attenuated MDA trajectory (22.4 nmol g^−1^ at day 21), indicating progressive and sustained oxidative protection rather than a one-time mitigation event. Pn in T4 declined sharply from day 7 onwards, reaching the lowest value of 9.1 µmol CO₂ m^−2^ s^−1^ at day 21, whereas T8 maintained Pn above 20 µmol CO₂ m^−2^ s^−1^ throughout the stress period. Proline dynamics showed that T8 accumulated osmolytes more rapidly and to a higher ceiling than T7 (Se NPs alone), indicating that biochar co-application accelerates the activation of proline biosynthesis pathways under stress, consistent with biochar-mediated enhancement of soil selenium bioavailability and subsequent amplification of selenium signaling cascades (Figure 10).

## Discussion

### Synergistic nature of combined drought–heat stress in wheat

The present study provides comprehensive quantitative evidence that concurrent drought and heat stress (DS+HS) imposes synergistic, supraadditive damage on wheat (cv. FBG-1800) across all measured parameters from whole-plant growth to subcellular gene expression. The 60.9% reduction in grain yield under T4, substantially exceeding the arithmetic sum of individual stress losses (DS: 42.4% + HS: 46.7% = 89.1% additive, but actual combined = 60.9% i.e., not additive in absolute terms but synergistic in relative impact per unit stress), confirms earlier observations that combined stresses do not simply superimpose individual responses but generate qualitatively distinct, interactively damaging physiological states^9,11^ The fundamental physiological conflict under concurrent DS+HS where heat demands stomatal opening for transpirational cooling while drought demands stomatal closure to conserve water^10^ was reflected in the paradoxical elevation of Ci despite reduced Pn and gs in T4, confirming that non-stomatal limitations (PSII photoinhibition, Rubisco thermal denaturation, and impaired electron transport) compound the stomatal CO₂ restriction under combined stress, as documented for wheat by Wahid et al. ^8^ and Lawlor and Tezara^40^.

The antioxidant enzyme collapse observed in T4 where SOD, POD, CAT, and APX all fell below control values despite individual stresses (T2, T3) inducing moderate enzyme upregulation constitutes one of the most mechanistically significant findings of this study. This oxidative overload phenomenon, wherein the rate of ROS generation surpasses the enzymatic scavenging capacity, has been proposed as a defining characteristic of combined stress biology.^9,12^ The 189.3% increase in H₂O₂ and 228.6% increase in MDA in T4 represent a level of oxidative damage consistent with large-scale membrane lipid peroxidation, protein carbonylation, and DNA strand breakage that would directly impair photosynthetic machinery, metabolic enzyme function, and reproductive development all contributing to the observed yield penalty.

### Mechanisms of Se NPs stress mitigation

Selenium nanoparticles applied to soil at 50 mg kg^−1^ (T5 and T7/T8) significantly enhanced stress tolerance through multiple convergent mechanisms. The substantial elevation of TaSOD1, TaCAT1, and TaAPX gene expression in Se NPs-treated plants under stress (T7: 4.86-, 4.32-, 3.98-fold respectively) demonstrates that selenium acts as a transcriptional activator of antioxidant defense, consistent with selenium's known role as a cofactor for glutathione peroxidase (GPX) and thioredoxin reductase (TrxR) enzymes that regenerate the ascorbate-glutathione cycle and maintain cellular redox homeostasis^12,41^ Nano-selenium's superiority over bulk ionic forms in plant bioavailability^43^ likely underlies the stronger responses observed here compared to earlier studies using selenate or selenite.^44^

The Se NPs-mediated activation of TaNHX1 (5.48-fold under T7) and associated improvement in Na⁺/K⁺ ratio homeostasis directly reflects selenium's capacity to modulate ion transporter expression through ABA-dependent signaling cascades. Selenium has been shown to activate SnRK2 kinases that phosphorylate and activate vacuolar Na⁺/H⁺ antiporters (NHX) and plasma membrane Na⁺ exclusion mechanisms (SOS pathway), thereby reducing cytosolic Na⁺ toxicity.^13,41^ Additionally, TaDREB2 upregulation (7.46-fold in T7) through Se NPs suggests activation of dehydration-responsive element binding (DREB) transcription factor networks that coordinately regulate proline biosynthesis, ABA biosynthesis, and stress-memory gene expression explaining the superior osmolyte accumulation observed. The Se NPs-mediated enhancement of plant Se content to 1.74 mg kg^−1^ DW in T7 (vs. 0.12 in T1) confirms bioavailable Se uptake from the Se NPs soil application, providing sustained antioxidant signaling throughout the stress period.

### Mechanisms of wheat straw biochar stress mitigation

Wheat straw biochar (2% w/w; T6) enhanced stress tolerance through mechanisms distinct from but complementary to those of Se NPs. The high BET surface area (186.3 m² g^−1^) and CEC (42.6 cmolc kg^−1^) of the produced biochar significantly improve soil water retention capacity by increasing micropore volume and hydrophilic functional groups that bind soil water under drought conditions.^16,17,45^ This soil physical effect directly mitigates the drought-induced component of combined stress evidenced by improved LRWC in T8 versus T7 at equivalent Se NPs application rates under DS+HS. Biochar's alkaline pH (9.1) also improves soil macronutrient availability, particularly phosphorus and potassium, in the slightly acidic sandy loam soil used, while its high K⁺ content provides a direct supplementary K⁺ source that counteracts drought-mediated K⁺ deprivation in the rhizosphere.

At the plant physiological level, biochar treatment independently enhanced chlorophyll content and Fv/Fm values under non-stress conditions (T6), suggesting activation of chloroplast protection mechanisms, possibly through biochar-mediated improvement in potassium and phosphorus availability that supports photosynthetic pigment biosynthesis and PSII membrane stabilization.^20,46^ The improved root morphology observed in T6 (root length: 23.8 cm vs. 22.6 cm in T1) may reflect biochar's positive effects on rhizosphere microbial communities, including mycorrhizal associations that enhance root architecture and nutrient acquisition.^18,47^

### Synergistic mechanisms of Se NPs + biochar co-application

The superior performance of Se NPs + biochar co-application (T8) over either amendment individually across all measured parameters provides strong evidence for true mechanistic synergy rather than simple additive effects. At least three distinct synergistic axes can be proposed from the integrated evidence.

First, biochar-mediated soil improvement amplifies Se NPs bioavailability. The enhanced soil water retention capacity conferred by biochar maintains the soil moisture content necessary for Se NPs dissolution, nanoparticle mobility in the soil matrix, and root uptake—processes that are severely compromised under drought-induced soil desiccation. Consistent with this, plant Se content in T8 (1.68 mg kg^−1^ DW) remained close to that of T7 (1.74 mg kg^−1^ DW) despite the additional water stress in the combined DS+HS treatment, indicating that biochar-buffered soil moisture sustained Se bioavailability and root uptake under conditions where Se NPs dissolution would otherwise be impeded by soil desiccation. The biochar-improved CEC also reduces Se leaching from the root zone, prolonging selenium availability throughout the stress period.

Second, Se NPs and biochar activate complementary antioxidant and osmotic pathways. While Se NPs primarily drive transcriptional antioxidant networks (*TaSOD1*, *TaCAT1*, *TaAPX* upregulation) and ionic homeostasis (*TaNHX1*), biochar primarily contributes through soil physical amelioration of the drought component and supplementary K⁺ supply. Together, they address distinct but interacting stress dimensions—Se NPs targeting the oxidative and molecular aspects of combined stress, while biochar ameliorates the water deficit component. This complementary targeting explains the substantially higher proline (96.2 vs. 78.4 µmol g^−1^ in T7) and glycine betaine (52.4 vs. 42.6 µmol g^−1^) accumulation in T8, indicating convergent activation of osmolyte biosynthesis pathways through multiple independent inputs.

Third, the pronounced *TaHSP70* upregulation in T8 (12.74-fold)—substantially exceeding even individual heat stress (T3: 6.84-fold)—suggests that the Se NPs + biochar system not only rescues the heat shock transcriptional response that is paradoxically attenuated under combined DS+HS, but further amplifies it. We propose that this amplification occurs through selenium-mediated enhancement of cellular redox balance and ABA signaling converging on heat shock transcription factor networks.^12,13^ It is further hypothesized that biochar-mediated restoration of soil water availability may support cellular turgor maintenance, thereby sustaining the ATP-dependent processes required for chaperone-assisted protein folding under heat stress. However, it must be explicitly acknowledged that this latter mechanistic link—between biochar-improved water status and energy availability for HSP70-mediated protein refolding—is not directly demonstrated by the data presented in this study. It is offered as a testable hypothesis that warrants future validation through targeted experiments combining turgor manipulation, ATP quantification, and HSP70 protein-level analyses under controlled conditions.

### Agronomic implications for Pakistani wheat production

The present study, conducted under wire house conditions simulating the agro-climatic stresses increasingly prevalent in Punjab's wheat belt, demonstrates that soil co-application of Se NPs (50 mg kg^−1^) and wheat straw biochar (2% w/w) can restore wheat grain yield to 88.0% of unstressed control values under combined drought-heat stress—a degree of recovery that compares favorably with currently available chemical or hormonal interventions. Several practical considerations merit attention for field-scale translation.

The green-synthesized Se NPs used here, produced via *Azadirachta indica* leaf extract, represent an environmentally benign production route that avoids toxic chemical reducing agents and yields biocompatible particles with favorable rhizosphere interactions.^22^ Regarding biochar, while wheat straw is abundantly available as a crop residue in Punjab's agricultural systems, it should be recognized that field-scale biochar production entails non-trivial processing costs, including pyrolysis equipment, fuel, grinding, sieving, and transportation. Characterizing biochar as a “zero-cost” input would therefore be an oversimplification. Economic analyses from South Asian contexts suggest that small-scale, on-farm pyrolysis units can substantially reduce per-unit costs compared to centralized production, and that the multi-season persistence of biochar's soil improvement effects distributes the initial investment across several cropping cycles^17,21^ Nevertheless, a rigorous cost-benefit analysis specific to Punjab's wheat production systems—accounting for Se NPs synthesis, biochar production, and application logistics at field scale—remains an important prerequisite before recommending broad adoption and is identified as a priority for follow-up research. Broader integration of such sustainable amendments into Pakistani wheat systems aligns with global priorities for climate-smart agriculture^48^ and will require supportive agronomic extension frameworks to facilitate farmer-level adoption.^49^

Future research priorities include: (i) field-scale validation across diverse wheat-growing agro-ecological zones of Pakistan with natural drought-heat co-occurrence; (ii) dose optimization studies for Se NPs (20–100 mg kg^−1^) and biochar (1%–4% w/w) to define the agronomically optimal and economically feasible application rates; (iii) evaluation of transgenerational stress memory effects—whether Se NPs + biochar pretreatment induces epigenetic stress memory in subsequent generations, an emerging area warranting systematic investigation; and (iv) life cycle assessment and cost-benefit analysis under farmer conditions in Faisalabad and surrounding districts.

## Conclusion

Combined drought–heat stress imposed synergistic physiological damage in wheat cv. FBG-1800, reducing plant height by 43.1%, grain yield by 60.9%, and net photosynthesis by 61.8%, while elevating H₂O₂ and MDA by 189.3% and 228.6%, respectively, alongside paradoxical antioxidant enzyme collapse. Soil co-application of Se NPs and wheat straw biochar (T8) produced genuine synergistic mitigation superior to either amendment alone, restoring grain yield to 88.0% and Pn to 89.9% of the unstressed control while reducing oxidative markers by 54%–59%, hyperactivating antioxidant enzymes (SOD: 2.14×; POD: 2.31×; CAT: 2.31×; APX: 2.09 × control), and dramatically upregulating stress-responsive genes—most notably TaHSP70 (12.74-fold) and TaDREB2 (9.38-fold)—thereby rescuing the paradoxically suppressed heat-shock response. PCA and Pearson correlation analyses confirmed that photosynthetic efficiency (*r* = 0.94 for Pn) and antioxidant capacity are the primary determinants of grain yield under combined stress, while the observed synergy operates through at least three mechanistic axes: biochar-amplified Se NPs bioavailability via soil moisture retention, complementary targeting of distinct stress components, and convergent Ca²⁺/ABA-mediated activation of HSP and DREB transcription factor networks. Collectively, these findings establish soil co-application of Se NPs and wheat straw biochar as a scientifically grounded, agronomically feasible, and environmentally compatible strategy for sustaining wheat productivity under combined drought–heat stress conditions projected to intensify across Pakistan's irrigated wheat systems, with field-scale validation and economic feasibility assessment identified as the next critical research priorities

## Data Availability

The datasets generated and/or analyzed during the current study are available from the corresponding author on reasonable request.
